# *Campylobacter* spp. isolated from poultry in Iran: Antibiotic resistance profiles, virulence genes, and molecular mechanisms

**DOI:** 10.1002/fsn3.3152

**Published:** 2022-11-21

**Authors:** Seyedeh Bita Mousavinafchi, Ebrahim Rahimi, Amir Shakerian

**Affiliations:** ^1^ Department of Food Hygiene, Faculty of Veterinary Medicine, Shahrekord Branch Islamic Azad University Shahrekord Iran; ^2^ Research Center of Nutrition and Organic Products, Shahrekord Branch Islamic Azad University Shahrekord Iran

**Keywords:** antimicrobial resistance, *Campylobacter*, poultry, quinolones

## Abstract

*Campylobacter* spp. genera is one of the most common causes of microbial enteritis worldwide. The objective of this work was to investigate the antimicrobial resistance (AMR) patterns, virulence genes, and genetic variation of thermophilic *Campylobacter* species collected from chicken meat samples in Iran. A total of 255 meat specimens were taken and transferred to the laboratory. Culture methods were utilized to identify the *Campylobacter* genus, and PCR and sequencing were performed to confirm the organisms. Antimicrobial susceptibility evaluation was performed using broth microdilution for six antimicrobials [ciprofloxacin (CIP), nalidixic acid (NAL), sitafloxacin (SIT), erythromycin (ERY), tetracycline (TET), and gentamicin (GEN)]. By using PCR, AMR and virulence genes were detected. The detection rate of *Campylobacter* spp. was 64 (25.09%) out of 255 meat samples, with *C. jejuni* and *C. coli* accounting for 41 (64.06%) and 14 (21.87%), respectively. Other *Campylobacter* isolates accounted for 14.06% of the total (nine samples). The antibiotic susceptibility of all *Campylobacter* isolates was tested using six antibiotics, and all (100%) were resistant to CIP and NAL. However, TET resistance was observed in 93.9% and 83.3% of *C. jejuni* and *C. coli* isolates, respectively. Four (8.2%) *C. jejuni* isolates were multidrug‐resistant (MDR), while none of the *C. coli* isolates were MDR. Two of the four MDR isolates were resistant to CIP, NAL, TET, and ERY, whereas the other two isolates were resistant to CIP, NAL, TET, and GEN. The values of the Minimum Inhibitory Concentration (MIC) were as follows: CIP, 64–256 μg/ml; NAL, 128–512 μg/ml; TET, 2–1024 μg/ml; SIT, 0.25–1 μg/ml; ERY, 1–32 μg/ml; and GEN, 1–256 μg/ml. *recR*, *dnaJ*, *cdtC*, *cdtB*, *cdtA*, *flaA*, *ciaB*, *cadF*, and *pidA* were discovered in more than 50% of *C. jejuni* isolates, although *wlaN*, *virbll*, *cgtB*, and *ceuE* were found in <50%. *flaA*, *cadF*, *pidA*, and *ciaB* were discovered in more than 50% of the *C. coli* samples, whereas *recR*, *cdtC*, *cdtB*, *cdtA*, and *cgtB* were found in less than half. For *C. coli*, the percentages for *wlaN*, *dnaJ*, *virbll*, and *ceuE* were all zero. The results of this study show *Campylobacter* isolates obtained from poultry have higher resistance to quinolones and TET, pathogenicity potential, and varied genotypes.

## Graphical abstract

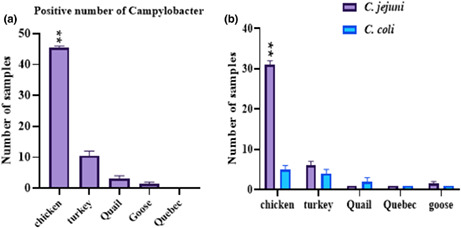

## 1 INTRODUCTION

*Campylobacter jejuni* and *Campylobacter coli* are foodborne pathogens that cause gastroenteritis (campylobacteriosis) in humans, affecting an estimated 95 million people each year throughout the world (Facciolà et al., 2017; Jonaidi‐Jafari et al., 2016; Torkan et al., 2018). *Campylobacter* infection is far more prevalent in affluent nations than foodborne diseases caused by *Listeria monocytogenes*, *Escherichia coli O157*, *Vibrio cholerae O139*, and *Shigella*, with an estimated 1.3 million foodborne cases annually in the United States alone (Hansson et al., 2018; Raissy et al., 2014; Rahimi et al., 2017). *C. jejuni* and *C. coli* are the major species causing sporadic gastroenteritis throughout the world (Li et al., 2020; Ommi et al., 2017). *C. jejuni* is responsible for 90% of campylobacteriosis cases (Facciolà et al., 2017; Kaakoush et al., 2015). *C. coli* comes second and accounts for 5%–10% of cases (Tam et al.,  2003). In 2018, 58% of *C. jejuni* outbreaks were linked to chicken and poultry products, according to reports (Tedersoo et al., 2022). The consumption of tainted poultry products is frequently associated with outbreaks. Poultry is considered a reservoir host of *C. jejuni* because this pathogen is found mostly in the gastrointestinal system of chickens and has been linked to meat contamination during processing (Beiranvand et al., 2022; de Melo et al., 2021). Poultry products are a popular source of proteins, vital amino acids, mineral salts, and vitamins for many individuals (Alagawany et al., 2021). The primary sources of human campylobacter infections are raw meat handling and uncooked meat‐eating. In controlled slaughtering procedures, chickens are slaughtered, skinned, and cut to bits by hand (Alagawany et al., 2021; Beiranvand et al., 2022; Cardoso et al., 2021). During the evisceration procedure, the corpse is drained, the visceral contents are separated, and the liver, heart, and intestines are gathered (Corry & Atabay, 2001). The discharge of digestive contents has the potential to infect these tissues. Since the demand for poultry products has increased in the world, ensuring their health and quality is the most important issue (Wickramasuriya et al., 2022). Food fraud, feces, microbiological contamination, physical faults, and product quality must all be examined regularly in poultry goods (Visciano & Schirone, 2021). A low *C. jejuni* prevalence (a percentage/proportion of colonized chickens in a flock) between chicken flocks or a 1 to 2 log10 reduction of *C. jejuni* loads in broiler intestines could lead to a decrease in public health risk, according to previous study's quantitative risk assessment and regression models (Pumtang‐On et al., 2021). As a result, lowering *C. jejuni* levels and preventing campylobacter colonization of chickens on farms are the most effective ways to limit the risk of campylobacter contamination of chicken meat (Hakeem & Lu, 2021). Biosecurity monitoring, feed additives, drinking water sanitation, and the use of bacteriophages, probiotics, and bacteriocins are among the strategies that researchers have attempted to develop and assess in primary broiler production so far (Gharaghie et al., 2022; Shehata et al., 2022). Even though several of these interventions have resulted in large decreases in *C. jejuni* burdens in chicken intestines, none of them have completely eradicated or prevented *C. jejuni* colonization in poultry (Hermans et al., 2011; Rahimi et al., 2014).

*Campylobacter jejuni* survival and virulence are mediated by motility, adhesion, quorum sensing, and biofilm formation. Although the importance of *C. coli* may seem small in percentage terms, the increasing rate of antibiotic resistance observed and the difference in risk factors for infection between the two species means that it is usually studied in the same way as *C. jejuni* (Li et al., 2020). While most *Campylobacter* infections induce mild diarrhea, severe, chronic, or systemic infections (such as bacteremia) can occur in young children, the elderly, and individuals with immunodeficiency disorders (Banisharif et al., 2019; Zarinnezhad et al., 2021). Under these circumstances, antibiotic therapy is required which may need the prescription of fluoroquinolone (FQ), macrolide, or aminoglycoside antibiotics (Dai et al., 2020). But *Campylobacter* has acquired multiple resistance mechanisms in response to antibiotic use in clinical settings and animal husbandry, and antibiotic‐resistant *Campylobacter* is becoming more common, compromising the efficacy of antibiotic therapy and creating a severe public health risk (Rossi et al., 2021). In Iran, there have been few studies on the prevalence and antibacterial resistance of intestinal Campylobacteriosis in people and poultry products. The lack of a broad monitoring program limited the availability of regular *Campylobacter* spp. culture supplies. Therefore, the present study aims to investigate the antimicrobial resistance (AMR) patterns, virulence genes, and genetic variation of thermophilic *Campylobacter* species collected from chicken meat samples in Iran.

## 2 MATERIALS AND METHODS

### 2.1 Collection of samples

Fresh chicken meat samples were collected from poultry farms, slaughterhouses, and restaurants located in Shahrekord city, the Islamic Republic of Iran in April 2020. A total of 255 meat specimens were taken and transferred to the laboratory under refrigeration (ice) within 1 h. A total of 255 samples of poultry meat including chicken (104 samples), turkey (71 samples), quail (35 samples), quebec (29 samples), and goose (16 samples) were collected.

### 2.2 Isolation and identification of *Campylobacter* spp.

The meats were put onto modified charcoal cefoperazone deoxycholate agar (mCCDA) (Oxoid Ltd.) including the *Campylobacter* mCCDA‐selective supplement, SR155E (Oxoid Ltd.) following arrival at the laboratory. CampyGenTM gas packets were used to create microaerophilic situations, which were maintained at 37°C for 48 h (Oxoid). *Campylobacter* colonies that are grayish, flat, wet, and spread easily were subcultured on Mueller Hinton Agar enriched with 5% defibrinated horse blood (MHS) and cultured at 37°C for 48 h under microaerophilic conditions. Isolates of *Campylobacter* were stored at 80°C in Mueller Hinton broth containing 25% glycerol (v/v).

### 2.3 Extraction of DNA and PCR identification of genus

The Qiagen QIAamp PowerFecal Kit (Qiagen) was used to extract genomic DNA from pure cultures, as directed by the manufacturer. Then, utilizing genus‐specific primers (C412F and C1228R), *C. jejuni* cj0414 gene primers (C1 and C3), and *C. coli* ask gene primers (CC18F and CC519R), a multiplex PCR was carried out (Yamazaki‐Matsune et al., 2007). The primers were chosen for their ability to distinguish between *Campylobacter* genus and species. 12.5 μl 2X Master Mix (Thermo Fisher Scientific), 1 μl primer (10 μM), 1.5 μl template DNA (20 μg/ml), and 7 μl sterile deionized water were used to make the PCR mixture (25 μl). The MiniAmp Plus Thermal Cycler was used to do one cycle of 95°C for 5 min, 35 cycles of 94°C for 45 s, 55°C for 45 s, and 72°C for 45 s, and a final extension of 72°C for 5 min (Applied Biosystems). Before being analyzed, the PCR products were kept at 4°C.

### 2.4 Antimicrobial susceptibility testing

Antimicrobial susceptibility was investigated by determining the Minimum Inhibitory Concentration (MIC) using the broth microdilution method. The isolates were evaluated against quinolones such as CIP, NAL (0.25–512 μg/ml), SIT (0.03–16 μg/ml); macrolide, ERY (0.06–64 μg/ml); and aminoglycosides such as GEN (0.06–64 μg/ml), and TET (0.125–1024 μg/ml). The antimicrobials were provided by Sigma‐Aldrich (St. Louis), except for SIT, which was bought from AdooQ BioScience (Irvine). In 0.1 N HCl and 70% ethanol, CIP and ERY were dissolved, respectively. Nalidixic acid and SIT were diluted in dimethyl sulfoxide, whereas GEN and TET were dissolved in water. Antibiotic solutions were filter‐sterilized before use, except for those dissolved in DMSO. Maintained *Campylobacter* samples were added onto MHS (Oxoid Ltd.) and cultured for 48 h under microaerophilic conditions at 37°C. To get well‐grown pure clusters devoid of glycerol, a subculture was carried out on the same medium and under the same circumstances. Suspensions equivalent to the 0.5 McFarland standard (1.5 × 10^8^ CFU/ml) were produced in normal saline for antimicrobial susceptibility testing, and the final concentration on a 96‐well plate was 2–5 × 10^6^ CFU/ml. The MIC was measured using a microplate reader (Synergy HT; BioTek Instruments Inc.) and verified by adding iodonitrotetrazolium chloride to 96‐well plates. The MIC was defined as the lowest antibacterial concentration that resulted in a significant reduction (>90%) in inoculum viability after 48 h. The MBC (minimal bactericidal concentration) was calculated. MBC was determined by the concentration at which no bacterial growth was detected after 48 h of incubation. Except for SIT, which lacks worldwide cut‐off values, the MIC values were evaluated according to the guidelines of the European Committee for Antibiotic Susceptibility.

### 2.5 Detection of AMR genes

Genes‐encoding AMR was determined by PCR experiments using primer in Table 1. The investigated genes were: *aphA‐3‐1* (GEN resistance), *cmeB* (efflux pump), *blaOXA*‐*61* (ampicillin (Beta‐lactam) resistance), *tet(O)* (TET resistance), and *aadE1* (aminoglycosides resistance; Table 1). After electrophoresis, bands of PCR products were observed under ultraviolet light using a Dual UV Transilluminator (Core BioSystem). The above‐mentioned approach was used to carry out the PCR procedure. After electrophoresis, bands of PCR products were visible under ultraviolet light using a Dual UV Transilluminator (Core BioSystem). The amplification products' bands were evaluated by comparing them to a 100‐bp DNA ladder (Dyne bio; Table 2). The AMPure XP beads (Beckman Coulter) were used to purify antibiotic‐resistant gene PCR products, which were then sequenced using the Sanger technique at SolGent (Solutions for Genetic Technologies).

**TABLE 1:** Primer sequences for the multiplex PCR experiment

Gene	Primer sequences (5′–3′)	Annealing temperatures (°C)	Product size (bp)	References
*16 S rRNA*	C412F: GGATGACACTTTTCGGAGC C1228R: CATTGTAGCACGTGTGTC	58	816	Linton et al. (1997)
*cj0414*	C‐1: CAAATAAAGTTAGAGGTAGAATGT C‐3: CCATAAGCACTAGCTAGCTGAT	56	161	Wang et al. (1992)
*ask*	CC18F: GGTATGATTTCTACAAAGCGAG CC519R: ATAAAAGACTATCGTCGCGTG	60	502	Linton et al. (1997)
*racR*	GATGATCCTGACTTTG TCTCCTATTTTTACCC	45	584	Hermans et al. (2011)
*dnaJ*	AAGGCTTTGGCTCATC CTTTTTGTTCATCGTT	46	720	Ziprin et al. (2001)
*wlaN*	TTAAGAGCAAGATATGAAGGTG CCATTTGAATTGATATTTTTG	46	672	Linton et al. (1997)
*virbll*	TCTTGTGAGTTGCCTTACCCCTTTT CCTGCGTGTCCTGTGTTATTTACCC	53	494	Datta et al. (2003)
*cdtC*	CGATGAGTTAAAACAAAAAGATA TTGGCATTATAGAAAATACAGTT	47	182	Hickey et al. (2000)
*cdtB*	CAGAAAGCAAATGGAGTGTT AGCTAAAAGCGGTGGAGTAT	51	620	Hickey et al. (2000)
*cdtA*	CCTTGTGATGCAAGCAATC ACACTCCATTTGCTTTCTG	49	370	Hickey et al. (2000)
*flaA*	AATAAAAATGCTGATAAAACAGGTG TACCGAACCAATGTCTGCTCTGATT	53	585	Datta et al. (2003)
*cadF*	TTGAAGGTAATTTAGATATG CTAATACCTAAAGTTGAAAC	45	400	Konkel et al. (1999)
*pldA*	AAGCTTATGCGTTTTT TATAAGGCTTTCTCCA	45	913	Ziprin et al. (2001)
*ciaB*	TTTTTATCAGTCCTTA TTTCGGTATCATTAGC	42	986	Ziprin et al. (2001)
*ceuE*	CCTGCTACGGTGAAAGTTTTGC GATCTTTTTGTTTTGTGCTGC	48.9	793	Gonzalez et al. (1997)
*cgtB*	TAAGAGCAAGATATGAAGGTG GCACATAGAGAACGCTACAA	49.9	561	Gilbert et al. (2000)
*tet*(O)	GCGTTTTGTTTATGTGCG ATGGACAACCCGACAGAAG	54	559	Pratt and Korolik (2005)
*cme*B	TCCTAGCAGCACAATATG AGCTTCGATAGCTGCATC	54	241	Olah et al. (2006)
*bla*_OXA‐61_	AGAGTATAATACAAGCG TAGTGAGTTGTCAAGCC	54	372	Obeng et al. (2012)
*aphA‐3‐1*	TGCGTAAAAGATACGGAAG CAATCAGGCTTGATCCCC	54	701	Obeng et al. (2012)

**TABLE 2:** *Campylobacter* spp. prevalence in various sample types

Type of meat	Number of samples	Positive number of C*ampylobacter*	Positive number of *C. jejuni*	Positive number of *C. coli*	Positive number of other species
Chicken	104	46	32	6	8
Turkey	71	12	7	5	–
Quail	35	4	–	3	1
Quebec	29	–	–	–	–
Goose	16	2	2	–	–
Collect	255	64	41	14	9

### 2.6 Detection of virulence genes

Polymerase chain reaction was conducted using particular primers for the genes linked with virulence (*recR*, *wlaN*, *cdtB*, *cdtA*, *cdtC*, *virbll*, *flaA*, *pidA*, *cadF*, *ciaB*, *ceuE*, *cgtB*, and *dnaJ*). 12.5 μl of 2X Master Mix (Thermo Fisher Scientific), 1 μl of primer (10 μM), 1.5 μl of template DNA (20 μg/ml), and 7 μl of sterile deionized water made up the PCR mixture (25 μl). The MiniAmp Plus Thermal Cycler was used to perform one cycle at 95°C for 5 min, 35 cycles at 94°C for 30 s, 55°C for 45 s, and 72°C for 45 s, and a final extension at 72°C for 5 min (Applied Biosystems).

### 2.7 Statistical analysis

All data were input into a Microsoft Excel Sheet (Microsoft Corp.) and analyzed with SPSS version 20 data analysis. The connections were evaluated using the Chi‐square test and logistic regression. A *p*‐value of <.05 was statistically significant for all experiments.

## 3 RESULTS

### 3.1 Prevalence of *Campylobacter* spp. in poultry meat

**FIGURE 1:**
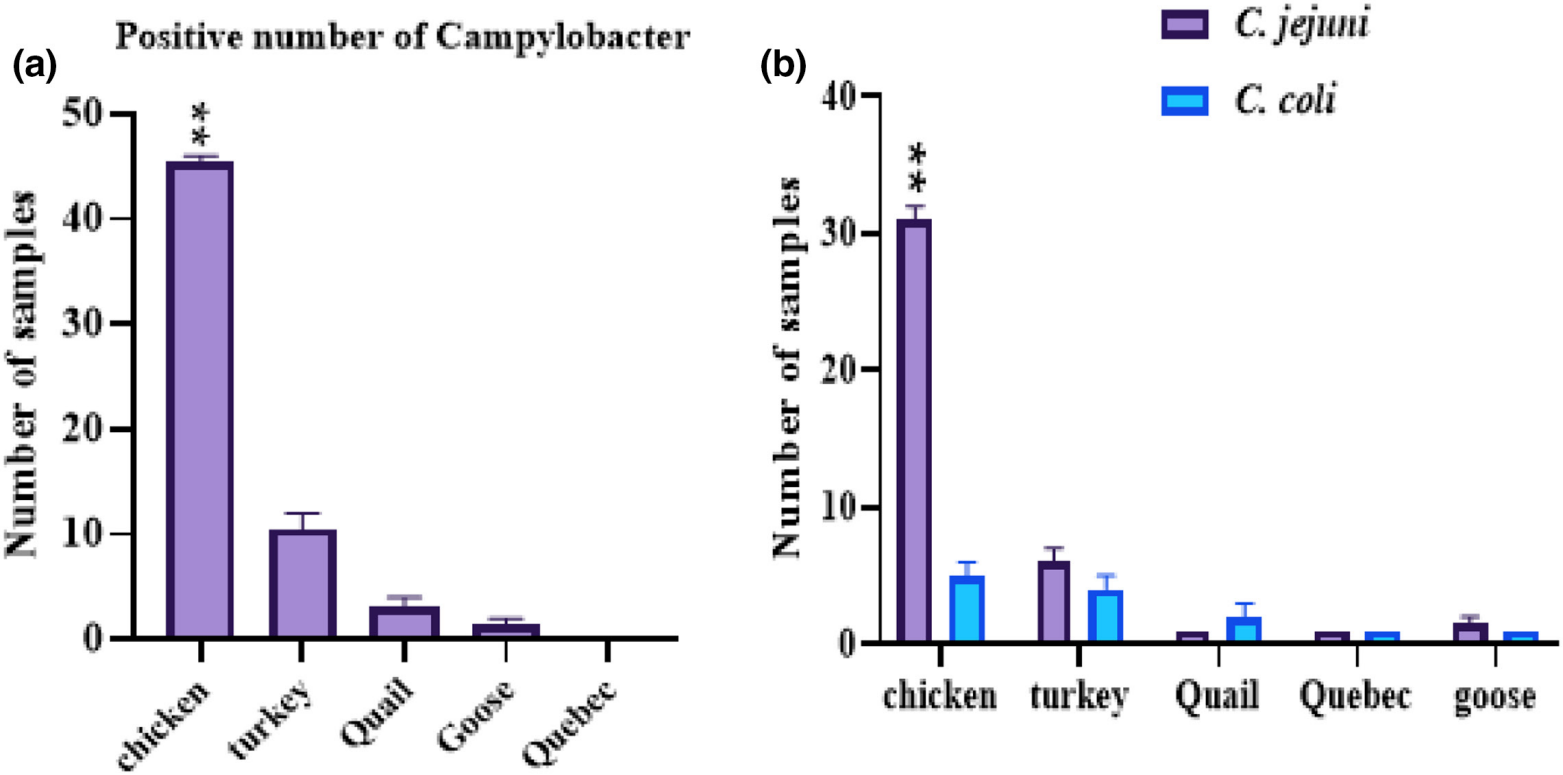
(a) *Campylobacter* spp. prevalence in various sample types. (b) *C. jejuni* and *C. coli* prevalence in various sample types

The detection rate of *Campylobacter* spp. was 64 (25.09%) out of 255 meat samples, with *C. jejuni* and *C. coli* accounting for 41 (64.06%) and 14 (21.87%), respectively. Other *Campylobacter* isolates accounted for 14.06% of the total (9 samples; Figure 1).

The highest rate of *Campylobacter* contamination was with chicken and turkey meat. Out of 104 samples of chicken meat, 46 samples (44.23%) were infected with *Campylobacter* spp. In the chicken meat, *C. jejuni* with 69.56% (32 samples) and *C. coli* with 13.04% (6 samples) had the highest distribution. Out of 71 turkey meat samples, 12 samples (16.90%) were infected with *Campylobacter*. The distribution of *C. jejuni* and *C. coli* in turkey meat was 58.33% (7 samples) and 41.66% (5 samples), respectively (Figure 1). Quebec meat was not infected with *Campylobacter*. Also, out of 16 goose meat samples, only two samples (12.5%) were infected, both of which (100%) belonged to *C. jejuni*. Out of 35 samples of quail meat, only four samples (11.42%) were infected, of which three samples (75%) belonged to *C. coli* (Table 2).

### 3.2 Susceptibility testing for antimicrobials

The antibiotic susceptibility of all *Campylobacter* isolates was tested using six antibiotics, and they all exhibited significant levels of resistance to CIP, NAL, and TET. Ciprofloxacin and NAL resistance were 100%, whereas TET resistance was 93.9% for *C. jejuni* and 83.3% for *C. coli* (Table 3). Four *C. jejuni* isolates (8.2%) were MDR, while none of the *C. coli* isolates were MDR. Two of the four strains isolated were CIP, NAL, TET, and ERY resistant, whereas the other two were CIP, NAL, TET, and GNT resistant. There was a 100% sensitivity to SIT, whereas 4.1% of the *C. jejuni* isolates were resistant to ERY and GEN (Table 4). Results showed that most of the *C. coli* isolates obtained from meat samples were resistant to at least three antibiotics. In fact, these isolates showed the MDR phenotype. There was a statistical difference between the specimens and AMR incidence (*p* < .05).

**TABLE 3:** Data on antimicrobial resistance in *Campylobacter jejuni* and *Campylobacter coli*

Antimicrobial	Species	Resistance (100%)	Number of isolates at the indicated minimal inhibitory concentration (μg/ml)										
			0.5	1	2	4	8	16	32	64	128	256	512
CIP	*C. jejuni*	100	0	0	0	0	0	1	36	10	2	0	0
	*C. coli*	100	0	0	0	0	0	0	4	2	0	0	0
SIT	*C. jejuni*	0	2	1	0	0	0	0	0	0	0	0	0
	*C. coli*	0	1	0	0	0	0	0	0	0	0	0	0
NAL	*C. jejuni*	100	0	0	0	0	0	0	1	25	17	6	0
	*C. coli*	100	0	0	0	0	0	0	0	1	5	0	0
ERY	*C. jejuni*	4.1	38	3	2	3	1	1	0	0	0	0	0
	*C. coli*	0	1	1	4	1	0	0	0	0	0	0	0
GEN	*C. jejuni*	4.1	27	8	0	0	0	0	0	0	0	0	0
	*C. coli*	0	1	3	0	0	0	0	0	0	0	0	0
TET	*C. jejuni*	93.9	2	1	0	0	0	0	3	10	22	10	1
	*C. coli*	83.3	0	0	0	0	0	0	0	1	2	2	0

**TABLE 4:** Antibacterial sensitivity profile of *Campylobacter jejuni* isolates

Type of meat	*C. jejuni*	*aphA‐3‐1*	*cmeB*	*tet(O)*	*blaOXA‐61*	*aadE1*
Chicken	1	−	−	+	−	+
	2	−	−	+	−	+
	3	−	−	−	+	−
	4	−	−	−	+	+
	5	−	−	−	−	−
	6	−	−	+	+	+
	7	−	+	+	+	+
	8	−	−	−	+	−
	9	−	−	+	−	+
	10	−	−	−	+	−
	11	−	−	+	−	+
	12	−	−	+	+	−
	13	−	−	+	−	+
	14	−	−	−	+	+
	15	−	−	−	+	−
	16	−	−	−	+	+
	17	−	+	+	+	−
	18	−	−	+	+	+
	19	−	−	+	+	−
	20	−	−	−	−	−
	21	−	−	−	−	−
	22	+	−	−	+	+
	23	−	−	−	+	−
	24	−	−	−	+	−
	25	−	−	−	−	−
	26	+	−	+	+	−
	27	−	−	+	−	+
	28	−	−	+	+	+
	29	−	−	−	+	+
	30	−	+	−	−	−
	31	−	−	+	−	+
	32	−	−	−	+	+
Turkey	1	−	−	+	−	+
	2	−	−	+	−	+
	3	−	−	−	+	−
	4	−	−	−	+	+
	5	−	−	−	−	−
	6	−	−	+	+	+
	7	−	+	+	+	+
Goose	1	+	−	+	+	−
	2	−	−	+	−	+
Quail	0	−	−	−	−	−
Quebec	0	−	−	−	−	−

The values of the MIC were as follows: CIP, 64–256 μg/ml; NAL, 128–512 μg/ml; TET, 2–1024 μg/ml; SIT, 0.25–1 μg/ml; ERY, 1–32 μg/ml; and GEN, 1–256 μg/ml.

Detection of antibiotic‐resistant genes showed that *tet(O)*, *blaOXA‐61* were present in all isolates. However, there were no bands for the multidrug efflux pump gene (*cmeB*) or the *aphA‐3‐1* gene in any of the strains. *blaOXA‐61* and *aadE1* genes were found to be most common in *C. coli* isolates (Table 5).

**TABLE 5:** Antibacterial sensitivity profile of *Campylobacter coli* isolates

Type of meat	*C. coli*	*aphA‐3‐1*	*cmeB*	*tet(O)*	*blaOXA‐61*	*aadE1*
Chicken	1	−	−	+	+	+
	2	−	+	−	+	+
	3	−	+	+	+	+
	4	−	−	+	+	+
	5	−	+	−	+	+
	6	−	+	+	+	+
Turkey	1	−	−	+	+	+
	2	−	+	+	−	−
	3	−	+	−	+	+
	4	−	−	−	−	−
	5	−	+	−	+	+
Quail	1	−	+	−	+	+
	2	−	−	−	−	−
	3	−	+	−	+	+
Goose	0	−	−	−	−	−
Quebec	0	−	−	−	−	−

PCR was used to confirm the existence of virulence genes *recR*, *wlaN*, *cdtB*, *cdtA*,*cadF*, *cdtC*, *virbll*, *flaA*, *pidA*, *ciaB*, *ceuE*, *cgtB*, and *dnaJ*. *recR*, *dnaJ*, *cdtC*, *cdtB*, *cdtA*, *flaA*, *ciaB*, *cadF*, and *pidA* were discovered in more than 50% of *C. jejuni* isolates, although *wlaN*, *virbll*, *cgtB*, and *ceuE* were found in <50% (Table 6). *flaA*, *cadF*, *pidA*, and *ciaB* were discovered in more than 50% of the *C. coli* samples, whereas *recR*, *cdtC*, *cdtB*, *cdtA*, and *cgtB* were found in less than half. For *C. coli*, the percentages for *wlaN*, *dnaJ*, *virbll*, and *ceuE* were all zero (Table 7).

**TABLE 6:** Distribution of genotypes amongst the *Campylobacter jejuni* strains isolated from different types of raw meat samples

Type of meat	Sample	*recR*	*dnaJ*	*wlaN*	*Virbll*	*cdtC*	*cdtB*	*cdtA*	*flaA*	*cadF*	*pidA*	*ciaB*	*ceuE*	*cgtB*
Chicken	1	+	+	−	−	+	+	+	+	+	−	+	−	−
	2	+	+	−	−	+	+	−	+	+	+	+	+	+
	3	+	+	−	−	+	+	+	+	+	−	+	−	−
	4	+	+	−	−	+	+	+	+	−	+	+	+	+
	5	+	+	−	−	+	+	+	+	+	−	+	−	−
	6	+	−	−	−	−	−	−	+	+	+	+	−	−
	7	+	+	+	+	+	+	+	+	+	+	+	−	−
	8	−	−	−	−	−	−	−	+	+	−	+	+	+
	9	+	+	−	−	+	+	+	+	−	+	+	+	+
	10	+	+	−	−	+	+	+	+	+	−	+	+	+
	11	+	+	−	−	+	+	−	+	+	+	+	−	−
	12	+	+	−	−	+	+	+	+	+	+	+	−	−
	13	+	+	−	−	+	+	+	+	+	−	+	−	−
	14	−	+	−	−	+	+	+	+	−	−	+	−	−
	15	−	+	−	−	+	+	−	+	+	+	+	+	−
	16	+	+	−	−	+	+	+	+	−	+	+	+	−
	17	+	+	+	+	+	+	+	+	+	−	+	+	+
	18	+	+	−	−	+	+	+	+	+	+	+	−	−
	19	+	+	−	−	+	+	+	+	+	+	+	−	−
	20	+	−	−	−	−	−	−	+	−	−	+	−	−
	21	+	−	−	−	−	−	−	+	+	+	+	+	−
	22	−	+	−	−	+	+	+	+	+	+	+	+	+
	23	+	−	−	−	−	−	−	+	+	−	+	−	−
	24	−	+	−	−	+	+	+	+	−	+	+	−	−
	25	+	−	−	−	−	−	−	+	+	+	+	−	−
	26	+	+	−	−	+	+	+	+	+	−	+	−	−
	27	+	+	−	−	+	+	+	+	−	+	+	+	−
	28	+	+	−	−	+	+	+	+	+	−	+	+	+
	29	+	+	−	−	+	+	−	+	+	+	+	−	−
	30	+	+	+	+	+	+	+	+	−	+	+	+	+
	31	+	+	−	−	+	+	+	+	+	−	+	−	−
	32	+	+	−	−	+	+	+	+	+	−	+	−	−
Turkey	1	+	+	−	−	+	+	+	+	+	−	+	−	−
	2	−	+	−	−	+	+	+	+	−	−	+	−	−
	3	−	+	−	−	+	+	−	+	+	+	+	+	−
	4	+	+	−	−	+	+	+	+	−	+	+	+	−
	5	+	+	+	+	+	+	+	+	+	−	+	+	+
	6	+	+	−	−	+	+	+	+	+	+	+	−	−
	7	+	+	−	−	+	+	+	+	+	+	+	−	−
Goose	1	+	+	−	−	+	+	+	+	−	+	+	+	+
	2	+	+	−	−	+	+	+	+	+	−	+	−	−
Quail	0	−	−	−	−	−	−	−	−	−	−	−	−	−
Quebec	0	−	−	−	−	−	−	−	−	−	−	−	−	−

**TABLE 7:** Distribution of genotypes amongst the *Campylobacter coli* strains isolated from different types of raw meat samples

Type of meat	Sample	*recR*	*dnaJ*	*wlaN*	*Virbll*	*cdtC*	*cdtB*	*cdtA*	*flaA*	*cadF*	*pidA*	*ciaB*	*ceuE*	*cgtB*
Chicken	1	−	−	−	−	−	−	+	+	+	−	+	−	−
	2	−	−	−	−	−	−	−	+	−	+	+	−	−
	3	−	−	−	−	−	−	−	+	+	−	+	−	+
	4	−	−	−	−	−	−	−	+	+	+	+	−	−
	5	−	−	−	−	−	−	−	+	+	−	+	−	+
	6	−	−	−	−	−	−	−	+	+	+	+	−	−
Turkey	1	−	−	−	−	−	+	−	+	−	+	+	−	−
	2	−	−	−	−	−	−	−	+	+	+	+	−	+
	3	−	−	−	−	−	−	−	+	−	+	+	−	+
	4	+	−	−	−	−	−	−	+	+	−	+	−	−
	5	−	−	−	−	−	+	−	+	+	+	+	−	+
Quail	1	+	−	−	−	−	−	−	+	+	−	+	−	−
	2	−	−	−	−	−	+	−	+	+	+	+	−	+
	3	−	−	−	−	+	−	−	+	−	+	+	−	−
Quebec	0	−	−	−	−	−	−	−	−	−	−	−	−	−
Goose	0	−	−	−	−	−	−	−	−	−	−	−	−	−

## 4 DISCUSSION

Poultry contamination with *Campylobacter* at the farm level typically impacts the entire commercial poultry system from farm to fork, necessitating appropriate treatments to prevent transmission from poultry to people (Lu et al., 2021; Wang et al., 2016). *Campylobacter* was found in 25.09% of cases, with *C. jejuni* (64.06%) outnumbering *C. coli* (21.87%). The diagnostic accuracy was somewhat greater than that reported earlier for layers in the United States (Novoa Rama et al., 2018) but lower than that previously reported for layers in the Netherlands (Schets et al., 2017) and Sri Lanka (Kalupahana et al., 2013). *C. jejuni* is the most common cause of human campylobacteriosis, according to the literature (Wei et al., 2014), and our findings back up this claim. Furthermore, there have been instances where *C. coli* was the dominant or only species identified (Silva et al., 2011; Vickers, 2017; Wei et al., 2014).

Several *Campylobacter* species showed significant levels of resistance to antimicrobial drugs (CIP, NAL, and TET), which is consistent with prior observations in Korea (Lee et al., 2017) and elsewhere (Elhadidy et al., 2018; Zhang et al., 2020). The use of CIP was outlawed in Korea in 2010 (Ku et al., 2011), although mass injection of chickens with FQs, particularly enrofloxacin, is still permitted (Jiang et al., 2008). Despite the lack of quinolone usage, quinolone‐resistant *Campylobacter* bacteria have been detected in Australia (Abraham et al., 2020). Although the fact that FQs are outlawed in numerous countries, resistant strains persist in bacterial communities, which might explain why they continue to be found in people and animals (Sproston et al., 2018). Considering the poultry manufacturing systems, monitoring methods, and geographical region, the link between the use of FQs in livestock farming and the increasing prevalence of AMR infections varies (Fan et al., 2018). The rising incidence of human campylobacteriosis in Asia, Europe, and America is thought to be fuelled in part by a worldwide presence of *C. jejuni* isolates resistant to quinolones transmitted through poultry (Oh et al., 2017). Strong resistance to TET was found, which is consistent with similar accounts from Korea (Lee et al., 2017), China (Zhang et al., 2020), and India (Kabir et al., 2015). Because of its low cost and ease of administration to animals through drinking water, TET is commonly used in the poultry and pig sectors (Jonker & Picard, 2010). SIT (MIC values: 0.125–1 mg/ml) was shown to be effective against all strains. SIT has unique properties not seen in other quinolones, such as a cyclopropyl circle with fluorine at R‐1 and a chloride substituent at R‐8, which may describe its remarkable potency (Changkwanyeun et al., 2016). Previous publications with a MIC of 0.25 mg/ml (Özmerdiven et al., 2015; Yabe et al., 2010) corroborate our findings. SIT is a contender for campylobacteriosis clinical testing (Özmerdiven et al., 2015), and is used to eradicate MDR *Helicobacter pylori* (Pohl et al., 2019). This might be a game‐changer, considering there are few other treatments for severe campylobacteriosis (Pavlova et al., 2020).

The strains in this investigation showed minimal resistance to ERY and GEN, which has been documented in other Asian countries such as Vietnam (Carrique‐Mas et al., 2014), and China (41). In China (Zhang et al., 2020), the United States (Tang et al., 2017), and Europe, resistance to ERY has been modest and consistent. The limited tolerance to ERY might be partially explained by delayed development of resistant isolates when treated for ERY, as well as decreased resistance variant survivability (Luangtongkum et al., 2012). Because GEN is used to treat systemic infections, resistance to it has been limited. The use of current antimicrobial drugs with caution, as well as initiatives to develop novel alternative treatment alternatives, might assist to slow the spread of AMR (Lynch et al., 2020).

The virulence of *Campylobacter* species depends on their virulome (Han et al., 2019). *recR*, *dnaJ*, *cdtC*, *cdtB*, *cdtA*, *flaA*, *ciaB*, *cadF*, and *pidA* were detected in more than 50% of *C. jejuni* isolates in this investigation, whereas *wlaN*, *virbll*, *cgtB*, and *ceuE* were found in <50%. The diagnostic accuracy was comparable to those reported in a recent report from Korea (Oh et al., 2017), but greater than those in South Africa (Otigbu et al., 2018; Pillay et al., 2020) and Chile (González‐Hein et al., 2013). Any strain of bacteria appears to require *cadF* in order to invade epithelial cells (Ramires et al., 2020). The cellular membranes phospholipase A (*pldA*) was found more in *C. jejuni* than in *C. coli*, corroborating South African research (Pillay et al., 2020), whereas our findings for *ciaB* are higher than those of a prior Korean investigation (Oh et al., 2017). In cellular models, the expression of *cadF* and *ciaB* aids *Campylobacter* adherence and internalization (Ramires et al., 2020). *C. jejuni* had a somewhat greater *csrA* detection accuracy than *C. coli*, although *csrA* was absent in *Campylobacter* isolates from South Africa (Otigbu et al., 2018). *ggt* was not found in any of our samples. Although the latter was reported to be just 5.5% in Chile (Otigbu et al., 2018), our findings contrast with Finland's high values (30.9%–43.2%; Gonzalez et al., 2009). The disparity might be explained by the intricacy of the colonization process, which involves many genes and the utilization of isolates from a single sampling location (González‐Hein et al., 2013). Moreover, MDR and virulent *C. jejuni* isolates were detected more frequently in the summer than in the winter, demonstrating that temperature has a role in the expression of certain genes (Kim et al., 2019). We also mention that numerous virulence genes are plasmid‐mediated, which might have an impact on their presence in various isolates. The virulence genes identified in this work had previously been found in *Campylobacter* strains isolated from people (Abraham et al., 2020; Kim et al., 2019), indicating that these *Campylobacter* isolates may be virulent enough to cause human illnesses.

In this study, *Campylobacter* isolates were characterized by the detection of certain resistance and virulence factors, which is limited to understanding the mechanisms of resistance and virulence. The results of whole genome sequencing analysis can determine the epidemiology and evolutionary pathways of *Campylobacter* spp to better tailor interventions to reduce cases of campylobacteriosis in Iran.

## 5 CONCLUSION

In conclusion, *Campylobacter* spp. collected from hens in this study showed significant antibiotic resistance and harbored various virulence and AMR genes. These strains could pose a risk to public health. The intensive use of antibiotics in chicken farming is to blame for the increase in MDR *Campylobacter* isolates. Antibiotic resistance in pathogenic bacteria can be reduced by monitoring antibiotic resistance in *Campylobacter* and by using antimicrobials appropriately in feed production.

## FUNDING INFORMATION

This research received no specific grant from any funding agency in the public, commercial, or not‐for‐profit sectors.

## CONFLICT OF INTEREST

The authors declare that they have no competing interests.

## ETHICAL APPROVAL

The research was extracted from the Ph.D thesis in the field of Food Hygiene, and was ethically approved by the Council of Research of the Faculty of Veterinary Medicine, Shahrekord Branch, Islamic Azad University, Shahrekord, Iran. Verification of this research project and the licenses related to sampling process were approved by Prof. Ebrahim Rahimi (Approval Ref Number MIC19817).

## Data Availability

All data analyzed during this study are included in this published article.
